# Factors associated with willingness to use mHealth interventions for medication adherence among people living with HIV attending a tertiary hospital in sub-Saharan Africa

**DOI:** 10.1371/journal.pone.0309119

**Published:** 2024-08-15

**Authors:** John Olujide Ojo, Tope Michael Ipinnimo, Aderonke Christiana Afolayan, Kayode Rasaq Adewoye, Olagoke Olaseinde Erinomo, Paul Oladapo Ajayi, Taofeek Adedayo Sanni, Oluyemi Aduke Ogundun, Jimlas Opeyemi Ogunsakin, Ayodeji Lawrence Oluwayemi, Grace Bukola Esan, Oluwaseun Omotola Omoyele, Olumide Temitope Asake, Ademuyiwa Adetona, Oluseyi Adedeji Aderinwale, Olanrewaju Kassim Olasehinde, Ireoluwa Oluwatomisona Adeniyi, Oluwadare Martins Ipinnimo, Austine Idowu Ibikunle

**Affiliations:** 1 Department of Community Medicine, Afe Babalola University, Ado-Ekiti, Nigeria; 2 Department of Community Medicine, Federal Teaching Hospital, Ido-Ekiti, Nigeria; 3 Department of Anatomic Pathology, Federal Teaching Hospital, Ido Ekiti and Department of Anatomic Pathology, Afe Babalola University, Ado Ekiti, Nigeria; 4 Department of Community Medicine, Ekiti State University, Ado-Ekiti, Nigeria; 5 Osun State Primary Healthcare Board, Osogbo, Nigeria; 6 South Atlantic Health Systems, Abuja, Nigeria; 7 Faculty of Basic Medical Sciences, Department of Public Health, Osun State University, Osogbo, Nigeria; 8 Department of Obstetrics and Gynaecology, Federal Medical Center, Abeokuta, Nigeria; 9 Department of Medicine, Afe Babalola University, Ado-Ekiti, Nigeria; 10 Department of Obstetrics and Gynaecology, Ekiti State University Teaching Hospital, Ado-Ekiti, Nigeria; University of Manitoba Faculty of Health Sciences, NIGERIA

## Abstract

**Introduction:**

There is increasing evidence in favor of enhancing adherence to antiretroviral therapy (ART) in people living with HIV (PLHIV) through mobile health (mHealth) assessment and intervention. The study aims to establish the willingness to adopt mobile phone technology to enhance adherence to ART among PLHIV.

**Methods:**

The Researchers adopted a cross-sectional survey. Systematic sampling was employed in selecting 237 PLHIV in the HIV clinic for adults at Ido-Ekiti’s Federal Teaching Hospital, Nigeria. Data collection was via a 33-item semi-structured questionnaire administered by the interviewer. Information collected via the questionnaire included details on ownership of mobile phone technology, its usage, and willingness to use it to improve adherence to HIV medication. Descriptive statistics coupled with multivariate regression was employed in analyzing data, with the level of significance at 5%.

**Results:**

The respondent’s had a mean ±SD age of 46.6 ±10 years. Most of the participants were female (77.6%), and have been on ART for over 2years (88.2%). The vast majority of study participants 233 (98.3%) owned a mobile phone. 168 (70.9%) of them were willing to embrace mHealth interventions on medication adherence. Some of the factors influencing the respondent’s willingness to receive the intervention were older age (OR = 0.05, 95%Cl:[0.01–0.24]), having formal education (OR = 7.12, 95%Cl:[3.01–16.53]), being diagnosed over 10years ago (OR = 15.63, 95%Cl:[3.02–80.83]) and previous use of phone to send text messages, record video, access the internet, send email and search the internet for health-related information (OR = 2.2, 95%Cl:[1.2–3.9]; OR = 1.8, 95%Cl:[1.0–3.2]; OR = 2.5, 95%Cl:[1.4–4.7]; OR = 2.7, 95%Cl:[1.2–5.5] and OR = 2.0, 95%Cl:[1.0–3.8]) respectively.

**Conclusion:**

Many of the PLHIV had a cellphone and expressed willingness on their part to use it in receiving reminders to take their medication. Older age, formal education and internet users were significantly more willing to get reminders to take their medication.

## Introduction

Mobile health (mHealth) refers to the adoption of portable electronic devices with software applications in the provision of health services and management of patient information [1]. With roughly 5 billion cellphone users across the world, there is growing recognition of the role that mobile technologies can play in health services, particularly in low- and middle-income countries. According to the World Health Organization (WHO), mHealth is an electronic health (eHealth) area availing health information and services through mobile technologies including mobile phones and personal digital assistant phones [1]. There is growing employment of mobile phone and internet-based technologies in disseminating health information and enabling the delivery of healthcare services [2].

Adhering to antiretroviral therapy (ART) i.e. taking HIV medicines every day and exactly as prescribed is crucial to achieving successful treatment outcomes at both individual and programmatic levels. An approximate adherence rate of at least 95% is necessary to avert rapid development of drug resistance and failure of treatment [3]. Treatment adherence is the extent to which one takes the prescribed medication. Achieving an undetectable viral load and avert drug resistance, persons on antiretroviral drugs must ensure they take at least 95% of doses prescribed at the recommended times [4,5]. Adherence constitutes a crucial predictor of HIV viral replication suppression, emergence of drug resistance, disease progression and death [4,5]. Monitoring and evaluation of adherence to ART are, as such, crucial tools for public health surveillance in the primary and secondary prevention of HIV and AIDS [6].

The highlighted reasons emphasize the need to identify better approaches to promoting adherence to ART regimens among patients. The growth in reach of wireless telecommunication networks in South Asia, sub-Saharan Africa, and other underserved regions of the world with a high prevalence of HIV, has enhanced the popularity of mobile phones as means of communication among the populace [7]. Additionally, there have been proposals of mobile phone technology interventions, as a method to improve adherence to ART among patients [8]. Using mHealth to ensure adherence among PLHIV dates back to about two decades during the escalation and emergency periods of the HIV epidemic in Nigeria. However, one of the major challenges experienced was the failure to provide correct phone numbers by the clients. One is not sure if this was done deliberately to prevent contact or an unwillingness of the client to be reached by the health system via phone for their care.

Robust research on the target population is necessary during the development of public health interventions to determine its willingness to be part of the program [9]. Less than 70% of PLHIV have expressed the desire for routine health-related electronic message notifications [10]. A study in Nigeria shows that more than 85% of the people possess a cellphone, out of whom, 28% own a smartphone [11]. This growing trend of smartphone ownership is quite promising to the efforts to deliver adherence interventions on a wide scale and cost-effectively, particularly in the case of those typically unable to access in-person interventions. Despite ART dramatically improving the patients’ health and reducing morbidity and mortality among HIV patients particularly in sub-Saharan Africa, there are still serious challenges concerning adherence to ART significantly contributing to drug resistance and ineffectiveness of treatment [12].

Several strategies have been tested in attempts to enhance the multipronged ART adherence challenge. However, there has been very little attention to the employment of mobile phone technology. Mobile phone ownership among ART patients is still limited with their level of willingness of to use mHealth interventions as medication reminders remaining unknown. Before implementing mobile phone technology-based treatment adherence strategies, the extent of willingness among patients to employ such interventions must be investigated. Therefore, this research assessed the ownership and utilization of mobile phone, willingness to use mHealth interventions as medication reminders, and establish the elements linked to these among patients on ART.

## Methods

### Study designs and setting

The study entailed a cross-sectional survey carried out between the months of January and April 2018 at the HIV clinic of Federal Teaching Hospital, Ido-Ekiti, Nigeria. The hospital is located in Ekiti State where it serves at least a million people. Ekiti State comprises 16 local government areas, and the major ethnicity is the Yoruba who co-exist with some Hausas, Igbos, and other ethnic groups from the country. In terms of occupation, the people are mainly farmers and traders in addition to civil servants and artisans. The main religions include Christianity, Islam, and African traditional religions. A tertiary hospital established in 1964 as a general hospital but attained the status of a teaching hospital in 2014. The HIV clinic was established in 2009 and supported by Institute of Human Virology of Nigeria. Nearly 1000 patients are currently on ART in the clinic which manages HIV infection in children, adults, and pregnant women.

### Participants, sample size determination and sampling technique

The study included all HIV patients who were 18 years or more and attended the adult HIV clinic of Federal Teaching Hospital, Ido Ekiti for their ART medication. We excluded those who declined consent or were too ill to respond. Determination of the sample size for the study was through the single population proportion formula: $n=\frac{z^{2}\times pq}{d^{2}}$ [13] with the following assumptions:

n = required minimum sample size,

Zα = 1.96 (standard normal deviation), 95% confidence level

d = 0.05 is the desired degree of accuracy

p = Percentage of patients on ART indicating willingness to embrace a mobile technology for

medication adherence [14]

q = 1-p, percentage of patients on ART unwilling to use a mobile technology for medication adherence

Finite correction for known population less than 10,000 using the formula below

$$n=\frac{n_{0}}{1+\frac{\left(n_{0}-1\right)}{N}}$$

Where;

n_0 =_ initial sample size estimate

n = minimum sample size adjusted for population <10,000

N = Total no of adult HIV positive clients enrolled in the teaching hospital (≈ 825)

The minimum sample size was estimated to be 237.

The study employed a systematic sampling technique in selecting the study participants and the total number of adult PLHIV on the clinic register was used as the sampling frame. A sampling interval of 3 was utilized to select eligible patients from the clinic. The first eligible participant was selected by balloting from the first 3 patients, others were subsequently selected by adding 3 to this.

### Data collection and study instrument

Selected eligible patients were approached and interviewed in a private section of the clinic by the research assistants after their clinic consultations using a paper-based pre-tested semi-structured interviewer-administered questionnaire. The research assistants were trained staff (community health extension workers) of the hospital who had experience with quantitative research data gathering. The questionnaire was adapted from the one developed and used by a previous study [14]. It was pre-tested among 25 PLHIV attending the HIV clinic of another teaching hospital located in another state of the country. Necessary adjustments were made to the questionnaire after the pre-test. Face and content validity were conducted by HIV experts, Pathologists, and Consultant Physicians from Ekiti State University Teaching Hospital, Ado-Ekiti and Federal Teaching Hospital, Ido-Ekiti.

The information obtained concerned ownership, mobile phone use, and willingness to use mobile phone technology to enhance adherence to HIV medicationsQuestions concerning ownership (number of phones, phone types, and the service plan used); access (such as the internet); use (including phone calls, text messages, social media, and software applications), and willingness to employ mobile devices in accessing and improving medical care were used. Some of the categories the questionnaire assessed include cell phone use: taking pictures; sending or receiving text messages; recording videos; sending or receiving email; accessing the internet; downloading applications; searching online for health information; and doing banking.

### Data and statistical analysis

The questionnaires were entered, cleaned and analyzed using the computer software SPSS (IBM SPSS Statistics for Windows, Version 25.0. Armonk, NY: IBM Corp.) in a password encrypted computer system. Descriptive and analytical statistics were used to summarize the data in tables. Frequencies and percentages, means and standard deviation (SD) were generated to summarize categorical and continuous data respectively. Chi-square test was used to detect statistically significant association between the willingness to use mHealth interventions to enhance medication adherence and independent patients’ variables. Yates continuity correction was employed where observation was less than 5 in two-by-two cross-tabulation. Binary logistic regression analysis was performed on factors significantly associated with willingness to employ mobile phone technology after the chi-square test to determine independent predictors of willingness to employ mobile phone technology in enhancing medication adherence among the respondents. P-values were considered significant at < 0.05 and odds ratios (OR) with 95% confidence interval were reported.

### Ethical consideration

Ethical approval (Protocol number: ERC/2017/11/20/1248) to carry out the research was sought from the hospital’s Ethical Review Committee. All participants were provided with the details of the study and their consent forms were signed (Written informed consent was obtained). Confidentiality was protected by not collecting identifiable information from the participants such as names and addresses. The collected data was used only for research purposes and thereafter stored in a passworded computer system with access only by the lead investigator.

## Results

As shown in Table 1 respondents age ranged from 28 to 68 years with a median (interquartile range) age of 45.0 (15.0) years. More than two-thirds (184; 77.6%) were female and about half (117; 49.4%) of the respondents were married. The majority were of Yoruba ethnicity (213; 89.9%), and employed (204; 86.1%) while 102 (43.0%) and 96 (40.5%) had attended at least a secondary or post-secondary school respectively. More than one-third (90; 38.0%) of the respondents were earning less than ₦10,000 monthly, 83 (35.0%) of them had their durations of diagnosis between five and seven years. Also, 17 (7.2%) of the respondents in this study smoke cigarette.

**Table 1:** Respondents’ socio-demographic characteristics.

Variable	Frequency (N = 237)	Percent (%)
**Age (in years)**		
Less than 40	52	21.9
40–49	94	39.7
50–59	68	28.7
60 and above	23	9.7
*Median (Inter-quartile range)*	*45*.*0 (15*.*0)*	
**Gender**		
Male	53	22.4
Female	184	77.6
**Marital Status**		
Single	17	7.2
Married	117	49.4
Separated/ Divorced	31	13.1
Widowed	72	30.4
**Ethnicity**		
Yoruba	213	89.9
Ibo	12	5.1
Hausa	3	1.3
Others	9	3.8
**Highest Education**		
No formal education	21	8.9
Primary School	18	7.6
Secondary School	102	43.0
Post-secondary School	96	40.5
**Occupation**		
Employed	204	86.1
Unemployed	33	13.9
**Monthly income (in Naira)**		
Less than 10,000	90	38.0
10,000–29,999	64	27.0
30,000–49,999	18	7.6
50,000–69,999	34	14.3
70,000 and above	31	13.1
**Duration of diagnosis (in years)**		
<2	22	9.3
2–4	47	19.8
5–7	83	35.0
8–10	50	21.1
>10	35	14.8
*Median (Inter-quartile range)*	*6*.*0 (5*.*5)*	
**Cigarette smoking**		
Yes	17	7.2
No	220	92.8

Table 2 shows that the majority (233; 98.3%) of the respondents in this study owned at least a phone; 131 (55.3%) had a cell phone while 133 (56.1%) had a smart phone. Among owners of mobile phone, 24.9% currently owned more than one device. Ninety-three (39.2%) had been utilizing phone(s) for between eleven to fifteen years and 120 (50.6%) were subscribing to data. Two hundred and thirty-three (98.3%) respondents were using their phone(s) to call out or receive phone calls, 163 (68.8%) also use their phones in sending or receiving text messages, 93 (39.2%) to access the internet, 85 (35.9%) for alarm, 75 (31.6%) to look for health information online.

**Table 2:** Mobile phone ownership and utilization among the respondents.

Variable	Frequency (N = 237)	Percent (%)
**Have a phone**		
Yes	233	98.3
No	4	1.7
**Number of phones**		
1	174	73.4
2	53	22.4
3	6	2.5
Didn’t own a phone	4	1.7
Type of phone*		
Cell phone	131	55.3
Smartphone	133	56.1
Didn’t own a phone	4	1.7
**Duration of phone usage (in years)**		
1–5	35	14.8
6–10	65	27.4
11–15	93	39.2
>15	40	16.9
Didn’t own a phone	4	1.7
**Subscribe to data**		
Yes	120	50.6
No	113	47.7
Didn’t own a phone	4	1.7
Reasons for using phone*		
Call out or receive phone calls	233	98.3
Pictures or video recording	114	48.1
Online banking	105	44.3
Send or receive text messages	163	68.8
Clock	120	50.6
Calendar/ Scheduling	84	35.4
Alarms	85	35.9
Calculator	105	44.3
Access the internet	93	39.2
Listen to music	86	36.3
Send or receive email	57	24.1
Check for health information online	75	31.6
Didn’t own a phone	4	1.7

Table 3 displays respondents’ willingness to use mobile phone to enhance medication adherence. About half (119; 50.2%) of the respondents had ever received health-related messages or calls and the major source of these messages/calls were from the ART/ Care & Support unit of the health facility (71; 30.0%). Furthermore, 168 (70.9%) expressed their willingness to receive medication and clinic appointment reminders through their mobile phones. About 30 (12.7%) did not want to receive any medication and clinic appointment reminders because they perceived it not necessary, while 24 (10.1%) did not want because they didn’t want people to know about their HIV status, 16 (6.8%) feel it was embarrassing and 12 (5.1%) considered it a disturbance. Also, 84 (35.4%) and 71 (30.0%) preferred to be reminded through text messages and voice calls respectively and when asked of the preferred time to be reminded 131 (55.3%) had no particular time that they preferred to be reminded.

**Table 3:** Willingness to use mHealth interventions to enhance medication adherence among respondents.

Variable	Frequency (N = 237)	Percent (%)
**Ever received health-related messages/ calls**		
Yes	119	50.2
No	118	49.8
The sender*		
The ART/ Care & Support Unit	71	30.0
WHO/ NGO	35	14.8
Network provider	29	12.2
Friends	20	8.4
Personal Physician	15	6.3
Spouse	15	6.3
Church	8	3.4
Can’t remember	4	1.7
Not applicable	118	49.8
**Willingness to receive medication reminder**		
Yes	168	70.9
No	69	29.1
Reasons for unwillingness*		
Don’t forget/ not necessary	30	12.7
Don’t want people around to notice	24	10.1
It is embarrassing	16	6.8
Consider it a disturbance	12	5.1
Don’t just like it	10	4.2
Not applicable	168	70.9
**Preferred medium to be reminded through**		
Text messages	84	35.4
Voice calls	71	30.0
Whatsapp	13	5.5
Not applicable	69	29.1
**How often would you like to be reminded**		
Anytime	53	23.6
Week of appointment	29	12.2
Weekly	8	3.4
Monthly	53	22.4
Bimonthly	22	9.3
Not willing	69	29.1
**Preferred time**		
Yes	37	15.6
No	131	55.3
Not willing	69	29.1
**When, if YES**		
Morning	5	2.1
Afternoon	13	5.5
Evening	19	8.0
Not applicable	200	84.4

Table 4 shows that age (p<0.01), highest education (p = 0.02), and duration of diagnosis (p<0.01) of respondents were significantly related to willingness on the patients’ part to adopt mobile phone technology in improving medication adherence.

**Table 4:** Factors Linked to willingness to use mHealth interventions to enhance medication adherence among respondents.

Variable	Willingness			
	Yes n = 168 (%)	No n = 69 (%)	Chi-square	p-value
**Age (in years)**				
< 40	45 (86.5)	7 (13.5)	24.59	**<0.01**
40–49	55 (58.5)	39 (41.5)		
50–59	57 (83.8)	11 (16.2)		
≥ 60	11 (47.8)	12 (52.2)		
**Gender**				
Male	40 (75.5)	13 (24.5)	0.70	0.40
Female	128 (69.6)	56 (30.4)		
**Marital Status**				
Single	10 (58.8)	7 (41.2)	1.41	0.70
Married	84 (71.8)	33 (28.2)		
Separated/ Divorced	23 (74.2)	8 (25.8)		
Widowed	51 (70.8)	21 (29.2)		
**Ethnicity**				
Yoruba	149 (70.0)	64 (30.0)	1.22	0.75
Ibo	10 (83.3)	2 (16.7)		
Hausa	2 (66.7)	1 (33.3)		
Others	7 (77.8)	2 (22.2)		
**Highest Education**				
No formal education	9 (42.9)	21 (57.1)	9.96	**0.02**
Primary School	12 (66.7)	6 (33.3)		
Secondary School	73 (71.6)	29 (28.4)		
Post-secondary School	74 (77.1)	22 (22.9)		
**Occupation**				
Employed	147 (60.9)	57 (39.1)	0.97	0.32
Unemployed	21 (63.6)	12 (36.4)		
**Monthly income (in Naira)**				
<10,000	58 (64.4)	32 (35.6)	6.42	0.17
10,000–29,999	52 (81.2)	12 (18.8)		
30,000–49,999	12 (66.7)	6 (33.3)		
50,000–69,999	26 (76.5)	8 (23.5)		
≥ 70,000	20 (64.5)	11 (35.5)		
**Duration of diagnosis (in years)**				
< 2	12 (54.5)	10 (45.5)	17.11	**<0.01**
2–4	32 (68.1)	15 (31.9)		
5–7	64 (77.1)	19 (22.9)		
8–10	28 (56.0)	22 (44.0)		
> 10	32 (91.4)	3 (8.6)		
**Cigarette smoking**				
Yes	13 (76.5)	4 (23.5)	0.06	0.80^a^
No	155 (70.5)	65 (29.5)		

^a^Yates’ Continuity Correction.

Table 5 shows the binary logistic regression for forecasters of willingness to use mHealth interventions to enhance medication adherence among respondent. After adjusting for confounder age, respondents’ highest education and duration of diagnosis were significantly connected to willingness to use mHealth interventions to enhance medication adherence. Respondents who were aged between 40–49 years (OR = 0.18, 95%Cl: [0.06–0.53]) and those aged 60 years and older (OR = 0.05, 95%Cl: [0.01–0.24]) were 5% and 18% times less likely to express willingness to use mHealth intervention to improve ART adherence compared those aged below 40 years old respectively. Also, respondents with a secondary (OR = 5.67, 95%CI: [2.80–13.65]) and tertiary education (OR = 7.12, 95%Cl: [3.01–16.53]) were 5.6 and 7.1 times respectively more likely to show willingness to embrace mHealth intervention to improve medication adherence compared to those lacking a formal education. Respondents diagnosed more than 10years ago had 15.6 times greater likelihood of being willing to use mobile phone technology to improve adherence to ART compared to those diagnosed less than 2years ago. (OR = 15.63, 95%Cl: [3.02–80.83]).

**Table 5:** Binary logistics regression for the predictors of willingness to employ mobile phone technology in promoting medication adherence among respondents.

Variable	AOR	95% C.I. for AOR		
		Lower	Upper	p-value
**Age (in years)**				
<40	1.00			
40–49	0.18	0.06	0.53	**<0.01**
50–59	0.49	0.14	1.69	0.26
≥60	0.05	0.01	0.24	**<0.01**
**Highest Education**				
No formal education	1.00			
Primary School	4.22	1.23	15.39	**0.04**
Secondary School	5.67	2.80	13.65	**0.01**
Post-secondary School	7.12	3.01	16.53	**<0.01**
**Occupation**				
Self-employed	1.00			
Employed	0.59	0.28	1.25	0.17
Unemployed	0.38	0.12	1.20	0.10
Retired	4.37	0.60	31.65	0.15
**Duration of Diagnosis**				
< 2	1.00			
2–4	3.11	0.87	11.05	0.08
5–7	2.47	0.78	7.84	0.13
8–10	1.82	0.51	6.53	0.36
>10	15.63	3.02	80.83	**0.01**

In Table 6, respondents that had been using their phones in sending or receiving texts, accessing the internet, sending and receiving emails, recording videos, searching for health-related information online, and those without phones were significantly willing to use mHealth interventions to enhance medication adherence. Respondents that had been using their phones for sending text messages had a 2.2 times higher likelihood of willingness to use mHealth interventions to enhance medication adherence than those that have not been using their phone to send or receive text messages. (OR = 2.2, 95% Cl: [1.2–3.9]) Those that had been using their phones for purposes of taking pictures or recording videos had a 1.8 times higher likelihood of willingness to use medication reminder interventions than those that have not been using their phone to take pictures or record video (OR = 1.8, 95%Cl: [1.0–3.2]). Also, respondents that had been accessing the internet and send email with their phones were 2.5 and 2.7 times more likely to be willing to receive medication reminder for better ART adherence compared to those that have not been using their phone to access the internet or send email (OR = 2.5, 95%Cl: [1.4–4.7]; OR = 2.7, 95%Cl: [1.2–5.5]) respectively. However, those without phones had a 3% less likelihood of willingness to use medication reminders to enhance their ART adherence compared to their counterparts lacking phones (OR = 0.03, 95%Cl: [<0.01–0.33]).

**Table 6:** Associations between respondent’s utilization of mobile phone and willingness to adopt mHealth interventions to enhance medication adherence.

Variable	Willingness				
	Yes n = 168 (%)	No n = 69 (%)	Chi-square	P	OR (95% CI)
**Reasons for using phone**					
Calling/receiving phone calls	167 (71.1)	66 (28.3)	4.15	**0.04**	7.6 (0.8–74.3)
Take picture or record video	88 (77.2)	26 (22.8)	4.23	**0.04**	**1.8 (1.0–3.2)**
Online banking	74 (70.5)	31 (29.5)	0.02	0.90	1.0 (0.5–1.7)
Sending/ receive text messages	124 (76.1)	39 (23.9)	6.81	**0.01**	**2.2 (1.2–3.9)**
Clock	87 (72.5)	33 (27.5)	0.31	0.58	1.2 (0.7–2.1)
Calendar/ Scheduling	63 (75.0)	21 (25.0)	1.08	0.30	1.4 (0.8–2.5)
Alarms	65 (76.5)	20 (23.5)	2.00	0.16	1.5 (0.8–2.8)
Calculator	74 (70.5)	31 (29.5)	0.02	0.90	1.0 (0.5–1.7)
Accessing the internet	76 (81.7)	17 (18.3)	8.71	**<0.01**	**2.5 (1.4–4.7)**
Listen to music	57 (66.3)	29 (33.7)	1.39	0.24	0.7 (0.4–1.3)
Sending/receiving email	48 (84.2)	9 (15.8)	6.46	**0.01**	**2.7 (1.2–5.8)**
Check for health information online	60 (80.0)	15 (20.0)	4.42	**0.04**	**2.0 (1.0–3.8)**
No phone	1 (25.0)	3 (75.0)	4.15	**0.04**	**0.03 (0.00–0.3)**

## Discussion

Most patients have access to mobile phones and can use them in enhancing HIV medication adherence interventions. Nearly all of the respondents in our study were in possession of a mobile phone, with about one-quarter currently being in ownership of more than one device. More than half have a smartphone. Access by patients to mobile phone in this study (98.3%) is concurrent with similar studies in the U.S.A where 96% [10] and 92.3% [15] had access to mobile phone. The similarity may be attributable to the rapid penetration of mobile phones in Nigeria’s urban centers.

The penetration of mobile phones in this study exceeded the figures recorded in other studies involving populations in developing countries where 81% were found in South Africa [16], 88.4% in China, 84% in Vietnam, 76.2% in Ethiopia [17] and 73.1% in South India [18]. The reason for the disparity may be differences in the ICT development index (IDI), ICT infrastructure, and the prevailing socioeconomic status in the identified countries creating a digital divide among countries [19]. The results point to high mobile phone ownership among patients on ART. For this reason, mobile phone-based interventions to enhance adherence to ART should be tested and further exploration conducted. The level of mobile phone technology access in this study is significantly higher than that recorded in the case of the general Nigerian population which is at 84% [20].

Text messaging constituted the most common mobile phone feature utilized by respondents. Even though 65.8% of the respondents said they sent or received a text message, there proportion was lower compared to the 80% of cellphone owners who were found to use their cell phone for text messaging [21]. Other popular activities were using the phone to accessing the internet and taking pictures or video. The percentage that owned a cellphone and took a picture or video (48.1%) were in-line with Pew estimates, that a 53% median among mobile phone owners report having done it within the last one year. The popularity of using mobile devices in taking pictures and videos is higher in South Africa (60%) compared to Nigeria (57%) [21].

Nearly three-quarters expressed their willingness to employ a mobile device to enhance their adherence to ART. The finding coincides with observations in other studies, which showed high willingness to receive medication reminder among PLHIV [22–24]. However, there are studies with different findings. For example, countries had higher figures including South Africa (96%) [16] and Peru (81%) [23], while lower figures were recorded in China (68.9%) [25], and North Carolina United State (33%) [26]. The discrepancy may be due to the differences in the educational status of the patients. This study indicated that patients with a secondary school education and above have a higher likelihood of willingness to receive medication reminders. Other studies similarly indicated literacy as constituting a major obstacle to the employment of mobile phone technology as medication reminder [23,27]. Patients who lack reading and writing abilities tend to prefer only voice calls from their healthcare providers. As such, an optional voice call or a recorded voice message would work better for them as a medication reminder intervention strategy. Our study availed further evidence supporting the willingness to employ mobile phones in reminding ART patient of their treatment. Implementation of the intervention among PLHIV in Nigeria would be critical. Even though Nigeria has four different major mobile service providers, it is not an barrier for mHealth intervention given that current communication technology ensures the compatibility of their platform with different mobile networks. Worth noting, is that people tend to change their mobile phone numbers frequently necessitating timely update of their contact information and confirmation of patient identity during the delivery of mHealth interventions [28].

## Conclusion

The vast majority of PLHIV have mobile phones and are willing to employ them to improve HIV medication adherence interventions. Older age, having formal education, duration of diagnosis, prior use of phone to send text or receive messages, access the internet to conduct an online search for health information and send mails constitute the most notable factors linked to the willingness among PLHIV to embrace mHealth interventions to enhance medication adherence. We recommend incorporation of mHealth interventions in the management of PLHIV as it is increasingly accessible to them and they have shown their openness to employing such strategies in managing their illness. The use of smartphone is increasingly becoming the norm for this population, with the potential of opening a new avenue for technology-based interventions via further data collection employing well-organized RCTs. Larger controlled studies are necessary to establish the intervention’s potential to improve not just ART adherence to ART, but also impact a wide range of relevant health outcomes for PLHIV.

## Supporting information

S1 File Minimal data set. (XLSX)
